# Development of Epitope-Blocking ELISA for Universal Detection of Antibodies to Human H5N1 Influenza Viruses

**DOI:** 10.1371/journal.pone.0004566

**Published:** 2009-02-24

**Authors:** Mookkan Prabakaran, Hui-Ting Ho, Nayana Prabhu, Sumathy Velumani, Milene Szyporta, Fang He, Kwai-Peng Chan, Li-Mei Chen, Yumiko Matsuoka, Ruben O. Donis, Jimmy Kwang

**Affiliations:** 1 Animal Health Biotechnology, Temasek Life Sciences Laboratory, National University of Singapore, Singapore, Singapore; 2 Department of Pathology, Singapore General Hospital, Singapore, Singapore; 3 Influenza Division, Centers for Disease Control and Prevention, Atlanta, Georgia, United States of America; 4 Department of Microbiology, Faculty of Medicine, National University of Singapore, Singapore, Singapore; University of Nebraska, United States of America

## Abstract

**Background:**

Human infections with highly pathogenic H5N1 avian influenza viruses have generally been confirmed by molecular amplification or culture-based methods. Serologic surveillance has potential advantages which have not been realized because rapid and specific serologic tests to detect H5N1 infection are not widely available.

**Methodology/Principal Findings:**

Here we describe an epitope-blocking ELISA to detect specific antibodies to H5N1 viruses in human or animal sera. The assay relies on a novel monoclonal antibody (5F8) that binds to an epitope comprising amino acid residues 274–281 (CNTKCQTP) in the HA1 region of H5 hemagglutinin. Database search analysis of publicly available sequences revealed that this epitope is conserved in 100% of the 163 H5N1 viruses isolated from humans. The sensitivity and specificity of the epitope-blocking ELISA for H5N1 were evaluated using chicken antisera to multiple virus clades and other influenza subtypes as well as serum samples from individuals naturally infected with H5N1 or seasonal influenza viruses. The epitope-blocking ELISA results were compared to those of hemagglutinin inhibition (HI) and microneutralization assays. Antibodies to H5N1 were readily detected in immunized animals or convalescent human sera by the epitope-blocking ELISA whereas specimens with antibodies to other influenza subtypes yielded negative results. The assay showed higher sensitivity and specificity as compared to HI and microneutralization.

**Conclusions/Significance:**

The epitope-blocking ELISA based on a unique 5F8 mAb provided highly sensitive and 100% specific detection of antibodies to H5N1 influenza viruses in human sera.

## Introduction

Highly pathogenic avian influenza (HPAI) H5N1 was first detected among poultry in China in 1996. One year later, H5N1 virus emerged in Hong Kong to cause fatal respiratory infections in humans [1]. Since then, HPAI H5N1 virus has continued to spread, causing outbreaks in birds and mammals in over fifty countries of the Old World [2]. Zoonotic H5N1 influenza transmission resulted in 387 confirmed human infections and 245 fatalities (as of August 2008) [3]. Indonesia has reported a high case fatality rate; 112 deaths among 137 cases of infection [3], [4]. Based upon the available epidemiologic surveillance data, the world is now in phase 3 (of 6) of a pandemic alert, according to the World Health Organization (WHO) [5], [6].

Serological investigations to detect specific antibodies from H5N1 infection or vaccination in humans and poultry are critical to the success of disease prevention and control programs. Several serologic tests are available, including hemagglutination inhibition (HI), microneutralization, enzyme linked immunosorbent assay (ELISA) and agar gel precipitation. However, HI tests have limited value for detecting antibodies against H5N1 viruses in humans and other mammals because of their low sensitivity, subtype cross-reactivity, and inter-assay variability [7], [8]. The microneutralization assay is currently recommended for detection of antibodies to HPAI H5N1 [9]. However, this test is labor-intensive and requires access to biocontainment facilities [10], rendering it impractical for rapid and high-throughput diagnostics [11]. The H5N1 ELISA (indirect ELISA) has been widely used in serologic surveillance of chicken and turkey flocks. However, cross-reacting antibodies elicited by infection or vaccination with seasonal influenza virus can yield false positive H5N1 test results that reduce the value of indirect H5N1 ELISA in humans [11].

We developed epitope-blocking ELISA (EB-ELISA) to detect serum antibodies to H5N1 viruses with high sensitivity and specificity. This assay yields a positive result when antibodies to the H5 hemagglutinin (HA) in test sera block binding of a labeled monoclonal antibody (mAb) to the H5 antigen. In practice, the intensity of the colored reaction product resulting from antigen-bound mAb is inversely proportional to the amount of epitope-specific antibody present in test serum [12], [13]. The biological significance of the test is determined by the epitope specificity of the mAb used in the EB-ELISA, and its broad applicability depends on the conservation of the epitope among H5N1 viruses. Further, the epitope recognized by the mAb should be highly antigenic so that antibodies to this epitope are consistently elicited in infected or vaccinated hosts. Here, we describe an EB-ELISA based on a mAb that meets these requirements. We evaluate the sensitivity and specificity of this method using experimental chicken anti-sera and demonstrate its advantages with H5N1 convalescent human sera.

## Materials and Methods

### Viruses

Human (n = 24) and avian (n = 2) influenza A viruses (subtype H5N1, clade 2.1) isolated in Indonesia were obtained from the Ministry of Health (MOH), Republic of Indonesia (Table 1). Influenza A viruses from other subtypes were obtained from the Agri-Food and Veterinary Authority (AVA) of Singapore (Table 1). A reassortant H5N1 virus containing the HA and NA from A/Vietnam/1203/04 (H5N1) in A/Puerto Rico/8/1934 (PR8) genetic background was described previously (14). High and low pathogenic viruses were propagated in the allantoic cavity of 11 day-old embryonated chicken eggs [15]. Virus titers were determined using a standard hemagglutination assay [16]. All viruses were clarified by centrifugation at 20,000×g for 20 min at 4°C, inactivated with formaldehyde as described previously [17] and stored at −80°C. Inactivated A/Indonesia/CDC669/2006 H5N1 virus was further concentrated by ultracentrifugation at 100,000×g for 2 h. All experiments with live viruses were performed in a biosafety level 3 (BSL-3) containment laboratory in compliance with CDC/NIH and WHO recommendations [18], [19] and approved by the Agri-Food and Veterinary Authority and Ministry of Health of Singapore.

**Table 1:** Avian influenza viruses used in this study.

Virus name
A/Indonesia/CDC7/06 a
A/Indonesia/CDC326/06 a
A/Indonesia/CDC329/06 a
A/Indonesia/CDC370/06 a
A/Indonesia/CDC390/06 a
A/Indonesia/CDC523/06 a
A/Indonesia/CDC594/06 a
A/Indonesia/CDC595/06 a
A/Indonesia/CDC597/06 a
A/Indonesia/CDC610/06 a
A/Indonesia/CDC623/06 a
A/Indonesia/CDC644/06 a
A/Indonesia/CDC669/06 a
A/Indonesia/TLL01/06 a
A/Indonesia/TLL02/06 a
A/Indonesia/TLL60/06 a
A/Indonesia/TLL177/06 a
A/Indonesia/TLL298/06 a
A/Indonesia/TLL485/06 a
A/Indonesia/TLL530/06 a
A/Indonesia/TLL535/06 a
A/Indonesia/TLL540/06 a
A/Indonesia/TLL561/06 a
A/Indonesia/TLL565/06 a
A/chicken/Indonesia/TLL101/06 a
A/duck/Indonesia/TLL102/06 a
A/chicken/Singapore/Sin/02 b
A/chicken/Singapore/Sin/92 c
A/common iora/Indonesia/F89/11/95 d
A/chicken/Singapore/Sin/98 e
A/mandarin duck/Singapore/Sin/93 f

The subtype of the virus used is indicated in superscript.

a H5N1.

b H3N2.

c H4N1.

d H7N1.

e H9N2.

f H10N5.

### Reverse genetics

The HA and NA genes of 17 viruses of H5N1 subtype from clades 1, 2, 4, 7 and 8 (Table 2) as well as subtypes H2N9, H6N8, H8N4, H12N5 (Table 2) were synthesized (GenScript, USA) based on sequences from the NCBI Influenza Database. The synthetic HA and NA genes were cloned into a dual-promoter plasmid for influenza A reverse genetics [14]. Reassortant viruses were rescued by transfecting plasmids containing HA and NA along with the remaining six influenza genes derived from A/Puerto Rico/8/34 into co-cultured 293T and MDCK cells using Lipofectamine 2000 (Invitrogen Corp). At 72 h post-transfection the culture medium was inoculated into embryonated eggs or MDCK cells. The HA and NA genes of reassortant viruses from the second passage were sequenced to confirm presence of introduced HA and NA genes and the absence of mutations.

**Table 2:** Reassortant influenza A viruses generated by reverse genetics.

Serial No.	Virus name (subtype)#	Clade	Host
1	A/HongKong/213/03* (H5N1)	1	Human
2	A/Vietnam/1203/04* (H5N1)	1	Human
3	A/muscovy duck/Vietnam/33/07 (H5N1)	1	Avian
4	A/Indonesia/CDC1031/07 (H5N1)	2.1	Human
5	A/turkey/Turkey1/05*(H5N1)	2.2	Avian
6	A/barheaded goose/Qinghai/12/05(H5N1)	2.2	Avian
7	A/Nigeria/6e/07(H5N1)	2.2	Human
8	A/muscovy duck/RostovonDon/51/07(H5N1)	2.2	Human
9	A/Egypt/0636-NAMRU3/07(H5N1)	2.2	Human
10	A/Anhui/1/05*(H5N1)	2.3	Human
11	A/Jiangsu/2/07 (H5N1)	2.3	Human
12	A/chicken/Nongkhai/NIAH400802/07 (H5N1)	2.3	Avian
13	A/VietNam/HN31242/07 (H5N1)	2.3	Human
14	A/Hongkong/156/97 (H5N1)	0	Human
15	A/chicken/Shanxi/2/06 (H5N1)	7	Avian
16	A/goose/Guiyang/337/06 (H5N1)	4	Avian
17	A/chicken/Henan/12/04 (H5N1)	8	Avian
18	A/duck/Nanchang/4-184/2000 (H2N9)	-	Avian
19	A/shorebird/DE/12/04 (H6N8)	-	Avian
20	A/duck/Yangzhou/02/05 (H8N4)	-	Avian
21	A/pintail/Alberta/49/03 (H12N5)	-	Avian

# Donor of HA and NA genes for derivation of PR8 reassortant viruses.

* Candidate vaccine strains for H5N1 virus vaccine development.

### Production of recombinant H5N1 HA0 protein (rHA0)

Viral RNA was extracted from allantoic fluid containing A/Indonesia/CDC669/06 (H5N1) using a commercial guanidium-phenol solution (Trizol Invitrogen, USA). The HA gene was amplified from reverse-transcribed RNA and cloned into pQE-30 vector (Qiagen, Germany) followed by transformation into *Escherichia coli* M15 pREP4 competent cells for protein expression (sequences of primers used for HA amplification are provided in supplementary file, Table S1 of the journal). Hexa-histidine-tagged fusion HA expression was induced by the addition of 1 mmol/L IPTG for 3 h and purified on a Ni-NTA column (Qiagen, Germany) [15].

### Production and characterization of mAbs

BALB/c mice were immunized twice 2 weeks apart by subcutaneous injection with 25 µg of rH5HA0 antigen mixed with adjuvant (SEPPIC, France). Mice received an additional intravenous injection of 25 µg of rH5HA0 antigen 3 days before the fusion of splenocytes with SP2/0 cells [20]. The fused cells were seeded in 96-well plates with selective medium, and their supernatants were screened by immunofluorescence assays as described below. Immunoglobulins from selected mAbs were isotyped using a commercial kit (Amersham Bioscience, England). Mouse mAb 5F8 was covalently conjugated to horseradish peroxidase (HRP) and purified from unbound enzyme using a commercial kit (Pierce EZ-Link plus Activated Peroxidase Kit).

### Immunofluorescence assay (IFA)

A recombinant baculovirus harboring the HA1-encoding region (amino acids 1–325) from the A/Indonesia/CDC669/06 HA gene was generated as described previously [21] for screening HA1-specific mAbs by IFA. Sf-9 and MDCK cells cultured in 96-well plates were infected with H5-HA1 recombinant baculovirus or with a panel of influenza viruses including Indonesian H5N1 strains and other non-H5 subtype viruses (Table 1). The cells were rinsed and fixed with 4% paraformaldehyde at 36 h (for Sf-9 cells) and 24–48 h (for MDCK cells) post-infection. Fixed cells were incubated with hybridoma supernatants at 37°C for 1 h, rinsed with phosphate buffered saline (PBS) and then incubated with a 1∶40 dilution of fluorescein isothiocyanate (FITC)-conjugated rabbit anti-mouse immunoglobulin (Dako, Denmark). Antibody binding was evaluated by wide-field epi-fluorescence microscopy (Olympus IX71) [22].

### Western blot

rH5HA0 expressing *E. coli* cultures were induced with IPTG, and cell pellets were lysed in sample buffer and separated on 12% SDS-PAGE. Proteins were then transferred onto nitrocellulose membrane and blocked with 5% non-fat milk in PBST (1× PBS and 0.1% Tween-20) for 1 h at room temperature. The membrane was incubated with hybridoma supernatant, rinsed with PBST and incubated with horseradish peroxidase (HRP)-conjugated rabbit anti-mouse IgG (Dako, Denmark) for 1 h at room temperature. Following washing with PBST, the membrane was developed using a chemiluminescent substrate (ECL Amersham Biosciences) as described previously [23].

### Epitope mapping of 5F8 mAb

Five partially overlapping fragments spanning the HA1-encoding region of the H5 HA gene of A/Indonesia/CDC669/06 were amplified by PCR and cloned into pQE-30 expression vector (sequences of primers used for fragment amplification are provided in the supplementary file, Table S2 of the journal). The fusion-peptides were expressed in *E. coli* as described above and analyzed by Western blot with anti-His antibody [23]. Eight additional truncated peptides (sub-fragment SF1–SF8, spanning amino acids 165–321; numbering for mature HA excluding signal peptide) were expressed as histidine-fusion peptides as described above and were used for Western blot analysis (sequences of primers used for fragment amplification are provided in supplementary file, Table S3 of the journal). A panel of fourteen mutants of sub-fragment SF4 with single mutations at amino acid residues 271–284 was produced by substituting each amino acid to alanine, except alanine-281, which was mutated to glycine. The point mutants were generated using the QuikChange Site-Directed Mutagenesis kit' (Stratagene, USA) following manufacturer's instructions (sequences of primers used for each amino acid substitution are provided in the supplementary file, Table S4 of the journal). These mutants were also expressed as His-fusion peptides in *E. coli* as described above.

### 5F8 epitope conservation analysis

In order to investigate the distribution of the 5F8 epitope among the 16 known HA subtypes, we compared 4143 full-length influenza A HA sequences from avian and human sources available in the NCBI Influenza Database (http://www.ncbi.nlm.nih.gov/genomes/FLU/Database/multiple.cgi) on August 26, 2008. The search was limited to “full-length sequences only” and identical sequences were removed. Alignment was performed using MUSCLE software to identify sequences with mutations within the 5F8 epitope.

### Experimental serum samples

Groups of White Leghorn chickens (n = 5), three weeks of age, were injected intramuscularly with each of 26 different inactivated H5N1 virus isolates from Indonesia (Table 1) or inactivated subtypes H3N2, H4N1, H7N1, H9N2 and H10N5 viruses emulsified in ISA-70 (SEPPIC, France) adjuvant. The injections were repeated twice at two-week intervals. In addition, 21 groups of chickens were immunized as indicated above with seventeen reassortant H5N1 viruses from clades 1, 2, 4, 7 and 8 (Table 2, No. 1-17) and four strains representative of subtypes H2N9, H6N8, H8N4 and H12N5 (Table 2, No. 18-21). Blood was collected 10 days after the 1^st^ immunization and 10 days after the 2^nd^ immunization. Sera were evaluated for antibodies against appropriate homologous strains by HI as described below.

### Human serum panels

Panels of human sera (n = 45) were divided into 3 groups. Group 1 comprised 10 human sera collected three to six months after infection with influenza H5N1, available from a serum repository of the MOH of Indonesia. Group 2 consisted of 15 serum samples from patients recovered from seasonal influenza A virus infections, available from the Repository of Singapore General Hospital (SGH), Singapore. Group 3 consisted of 20 serum samples from healthy volunteers with a history of no clinical influenza-like illnesses and no influenza vaccination, obtained from repositories of the MOH, Indonesia. The influenza exposure status of patients was documented in medical records of the MOH of Indonesia; individual identifiers were anonymized in compliance with applicable human subjects protection regulations.

### Epitope-Blocking ELISA

Optimal dilutions of purified H5N1 viral antigen or recombinant HA0 and mAb were determined by checkerboard titration to yield sub-saturating levels of the mAb. U-bottomed 96-well ELISA plates were coated with purified recombinant HA of A/Indonesia/CDC/669/06 prepared as described above (500 ng/well) or inactivated concentrated H5N1 virus (10 µg/well) and incubated overnight at 4°C in coating buffer (0.1 mol/L carbonate/bicarbonate, pH 9.6). Antigen-coated plates were washed with PBS (pH 7.5) containing 0.05% Tween 20 (PBST) and non-specific sites were blocked with 100 µL blocking buffer (PBS containing 5% skim milk) for 40 min at 37°C. Test serum samples were serially diluted two fold in PBST, and 100 µL was added to each well and incubated for 45 min at 37°C. The wells were rinsed four times with PBST and incubated with ∼120 ng of HRP-conjugated anti-H5 mAb 5F8 in 100 µL PBST for 1 h at 37°C. The wells were rinsed with PBST and incubated with 100 µL of 3, 3′, 5, 5′-tetramethyl benzidine (TMB, Sigma, USA). The reaction was stopped by adding 0.1N Sulfuric acid and the optical density (OD) determined at 450 nm using a multiwell plate reader.

The OD intensity reduction caused by serum antibodies blocking mAb binding was calculated for each sample dilution by using the formula: % inhibition = [(negative reference serum OD−test serum OD)/(negative reference serum OD−positive reference serum OD)]×100%. To determine the cut-off value, 126 specific pathogen-free chicken sera were obtained from the Animal Health Biotechnology Serum Bank, Temasek Life Sciences Laboratory, Singapore.

### Hemagglutination inhibition assay

Hemagglutination inhibition assays were performed as described previously [24]. Briefly, sera were treated with receptor destroying enzyme (RDE), serially diluted (2 fold) in V-bottom 96-well plates and then mixed with a standard amount of virus. Plates were incubated for 30 min at room temperature, and 1% chicken red blood cells were added to each well. The hemagglutination inhibition endpoint was the highest serum dilution in which agglutination was not observed.

### Microneutralization assay

Serum neutralizing antibody titers were determined by a multiwell plate cell culture assay as previously described [17]. Briefly, MDCK cells were seeded at 1×10^4^ cells/well in 96-well culture plates and cultured at 37°C in 5% CO_2_ to form a monolayer. Serial two fold dilutions of RDE treated serum samples were mixed separately with 100 TCID_50_ of A/Indonesia/CDC669/06 (H5N1) and incubated at room temperature for 1 h. The mixture was added to monolayer of MDCK cells in triplicate wells. The plates were incubated for 2 days at 37°C in DMEM supplemented with 1 µg/ml of TPCK-treated trypsin. The neutralizing titer was assessed as the highest serum dilution in which no cytopathic effect was observed by light microscope.

## Results

### Characterization of mAbs

**Figure 1:**
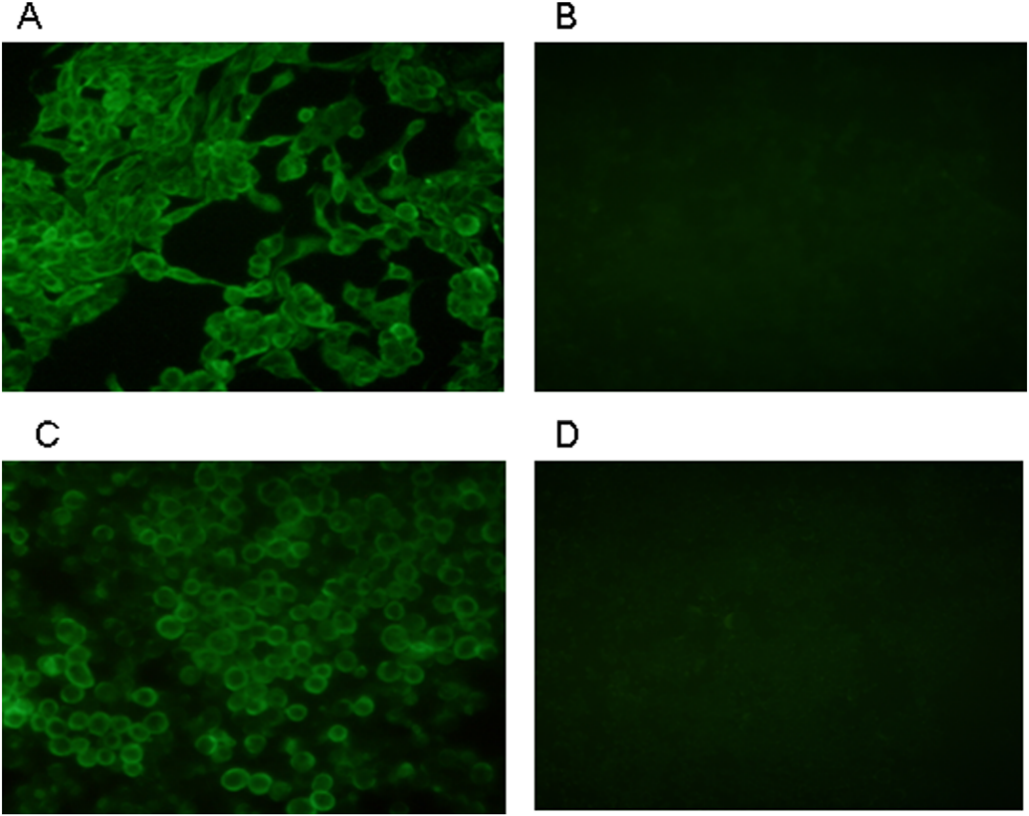
Monoclonal antibody characterization by immunofluorescence assay. Indirect immunofluorescence assay with cells infected with H5N1 virus or recombinant baculovirus expressing H5 HA. Infected cells were fixed and stained with mAb 5F8 and FITC-conjugated goat anti-mouse immunoglobulin. MDCK cells infected with A/Indonesia/CDC594/06 fixed at 12 h post-infection were stained with 5F8 (A); Sf9 insect cells infected with recombinant baculovirus expressing H5 HA1 were stained with 5F8 (B); mAb 5F8 did not bind to MDCK cells infected with A/common iora/Indonesia/F89/11/95 (H7N1) (C); and also failed to bind to Sf-9 cells infected with wt baculovirus (D).

Seven hybridomas secreted antibodies that bound to H5 HA1 expressed in recombinant baculovirus-infected insect cells and to H5N1 virus-infected MDCK cells by IFA (data not shown) were isolated. On the basis for lack of cross-reactivity with H1N1 and H3N2 virus-infected cells, we selected one hybridoma, 5F8 for further characterization. This hybridoma was biologically cloned twice by limiting dilution before production of mAb-containing cell culture fluid for the present study. mAb 5F8 yielded cytoplasmic immuofluorescence in MDCK cells infected with A/Indonesia/CDC594/06 (H5N1) virus (Fig. 1A) and Sf-9 cells infected with H5-HA1 recombinant baculovirus (Fig. 1C). In contrast, no fluorescence signal was observed in MDCK cells infected with an H7N1 virus (Fig. 1B) and viruses from 4 other subtypes listed in Table 1 (results not shown). Neutralization assays indicated that 5F8 does not affect the infectivity of A/Indonesia/CDC669/06 (H5N1) (data not shown). The sensitivity and specificity profiles of mAb 5F8 established by IFA indicated that this mAb met the requirements for epitope mapping and further EB-ELISA development. The 5F8 mAb was identified as IgM class.

### Epitope mapping of mAb 5F8

**Figure 2:**
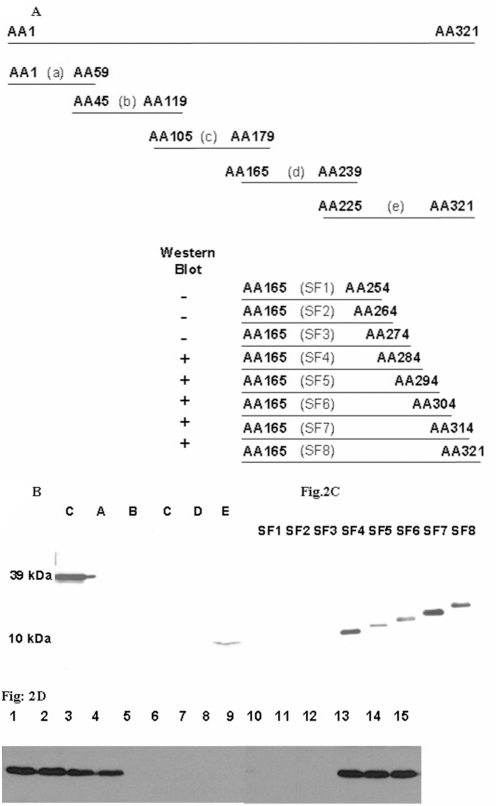
Mapping the mAb 5F8 epitope. (A) Fragments of the H5 HA coding region (termed “a” through “e” and SF1 to SF8; their boundaries indicated by N terminal and C terminal codon positions in HA) were expressed as histidine N-terminal-tagged fusion proteins. mAb 5F8 binding to fragments “a” through “e” (B) and sub-fragments SF1 to SF8 (from fragments “d” and “e”) in Western blot (C). +: rH5HA1 protein used as control. Western blot analysis of histidine-tagged HA sub-fragments (SF3 and SF4) with single amino acid substitutions revealed that binding by mAb 5F8 depends on the presence of specific amino acids spanning positions 274–281 (D).

The 5F8 mAb recognized the HA1 domain of H5 by Western blot assays (data not shown), suggesting that the epitope is either linear or readily refolded through short-range interactions. This property was exploited to map the HA epitope targeted by 5F8. To this end, partially overlapping expressed HA fragments were probed by Western blot analysis (Fig. 2A). mAb 5F8 reacted exclusively with fragment “e” (amino acids 225–321 of mature HA) (Fig. 2B). A panel of C-terminal nested deletions (SF1 through SF8) generated to refine the epitope map revealed that 5F8 mAb reacted with fragments SF4–SF8, indicating that the epitope comprised amino acids 274 to 284 (Fig. 2A and 2C). Subsequent analysis of a panel of expression constructs with single amino acid changes analyzed by Western blot indicated that 5F8 bound to mutants Y271A, G272A, N273A, M282A, G283A and A284G. In contrast, 5F8 failed to bind mutants C274A, N275A, T276A, K277A, C278A, Q279A, T280A, and P281A by Western blots (Fig. 2D). These results suggested that amino acids 274–281 (CNTKCQTP) were required for mAb 5F8 binding.

### Epitope analysis

Full length HA sequences from influenza A virus strains (n = 4143) were obtained from the Influenza Virus Database maintained by the National Center for Biotechnology Information. The available HA sequences from H5N1 viruses isolated from humans (n = 163) revealed that the CNTKCQTP epitope recognized by 5F8 mAb is conserved in all these viruses whereas the conservation rate is 96.9% among the additional 906 H5N1 viruses isolated from avian sources. Of the 162 H5 viruses with NA subtype other than N1 (H5N2-N9), the 5F8 epitope is present in 54.3% of the viruses (Table 3). Thus, the conservation rate of the 5F8 epitope among all avian H5 viruses is 91.7%. This epitope was absent among the HAs of subtype H2, H3, H4, H6-16 viruses (n = 2302). Although the epitope was present in 14 of the 610 H1 subtype viruses (approximately 2.3%), 13 of these viruses were isolated before 1991 and the last one in 1999 (data not shown). On the other hand, HA sequences of all H5N1 viruses used in this EB-ELISA study were found to have the CNTKCQTP epitope.

**Table 3:** Frequency of Monoclonal antibody 5F8 epitope among HA subtypes.

HA Subtype	Virus Host	5F8 epitope/total number of sequences	Conservation rate (%)
**H5N1**	Human	163/163	100%
**H5N1**	Avian	878/906	96.9%
**H5Nx (x = 2, 3, 5–9)**	Avian	88/162	54.3%
**H1**	Avian, human	14/610	2.3%
**H3, H4, H6, H7, H8, H9, H10, H11, H12, H13, H14, H15 and H16**	Avian, human	0/2302	0%

### Antisera to HA subtypes and H5N1 clades

A panel of antisera to influenza A viruses from various subtypes was developed to determine the specificity of mAb 5F8 in an epitope-blocking assay. Viruses from H5N1 clades or HA subtypes that were not available in our laboratory were rescued by reverse genetics as reassortants with the six internal genes (PB2, PB1, PA, NP, M and NS) from A/Puerto Rico/8/34. Seventeen H5N1 reassortant viruses harbouring HA and NA of H5N1 viruses from clades 0, 1, 2.1, 2.2, 2.3, 4, 7, and 8 were generated (Table 2). The newly derived clade 1, 2.2 and 2.3 H5N1 reassortants included strains recommended by WHO for vaccine development (Table 2) [25]. Four additional subtypes, H2N9, H6N8, H8N4 and H12N5, were also generated as reassortant viruses. These viruses were subsequently used to immunize chickens and produce antisera to evaluate the EB-ELISA. The initial characterization of the immune response of chickens to the homologous viruses was characterized by HI tests (Table 4).

**Table 4:** EB-ELISA with immunized chicken sera.

Chicken serum against (n = 5/group)	Day 10 after 1^st^ Immunization		Day 10 after 2^nd^ Immunization	
	HI titer#	EB-ELISA*	HI titer#	EB-ELISA*
26 Indonesian H5N1 strains from clade 2.1 (n = 5/group)	11.79±0.98 a	52.67±3.10	84.67±10.40	334.67±25.40
ck/Sg/02 (H3N2)	3.5±0.50	0	6.0±1.55	0
ck/Sg/92 (H4N1)	0	0	0	0
ck/Sg/94 (H7N1)	0	0	0	0

# Against H5N1 strain A/Indonesia/CDC669/06.

* Highest serum dilution blocking >30%.

a Geometric mean±Standard Error (SE).

### Development of an epitope-blocking (EB) ELISA assay

Serum antibodies to the H5 HA can be detected by virtue of their ability to block the binding of a mAb to the target epitope in an ELISA assay. To develop this assay, we relied on serum panels from normal and influenza-immunized chickens. First, a panel of 126 normal chicken serum samples lacking antibodies to influenza virus was used to determine the baseline of non-specific reduction in 5F8 binding to H5 antigen in the EB-ELISA. Mean reduction of EB-ELISA readings (i.e. blocking) was 8.5% for this serum panel, with a standard deviation (SD) of 9.3. Specific blocking activities can be determined with 95% confidence if a “cut-off value” of ≥30% is set for chicken serum samples. The latter was obtained by adding 2 SD to the mean 8.5% blocking (8.5+18.6 = 27.1%).

**Figure 3:**
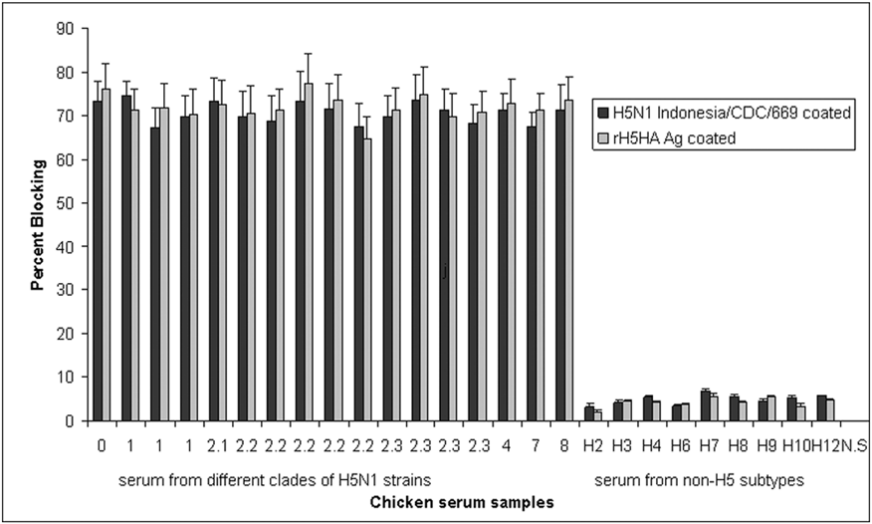
Sensitivity and specificity of the EB-ELISA. Performance of the EB-ELISA with sera from chickens immunized with different clades of H5N1 and other HA subtypes. Chicken sera collected 10 days after second immunization were adjusted to HI titer of 16 and subsequently diluted 5-fold for analysis in EB-ELISA. Antisera to H5N1 clade 0, 1, 2.1, 2.2, 2.3, 4, 7 and 8 and to other subtypes such as H2N9, H3N2, H4N1, H6N8, H7N1, H8N4, H9N2, H10N5 and H12N5 were analysed. Inhibition above the cut-off value of >30% blocking was considered as positive; i.e. antibodies to H5N1 were present. The results were expressed as the arithmetic mean of percent blocking values (n = 5/group, whiskers above bars represent the standard error of the mean). NS: Normal preimmune chicken serum.

The sensitivity and specificity of the mAb 5F8-based EB-ELISA was investigated using a panel of antisera from experimentally immunized chickens. Chicken sera collected 10 days after the 2^nd^ immunization were first diluted to obtain HI titer of 16 to the homologous virus to normalize antibody concentrations prior to use in EB-ELISA. The EB-ELISA endpoint of the HI-normalized sera was then determined by further analysis of log2 serial dilutions. Sera from chickens immunized with H5N1 reassortant influenza viruses (Table 2) yielded mAb 5F8 blocking values above the 30% cutoff in EB-ELISA (Fig. 3). Many samples showed >65% inhibition at dilutions well below detection by HI titer (<4) indicating that the EB-ELISA detected low levels of antibody to H5 (Fig. 3). Moreover, sera from chickens immunized with H1–H4, H6–H10 and H12 showed maximum blocking of <10%, well below the 30% threshold established for samples containing specific antibodies (Fig. 3). These results indicate that the EB-ELISA could positively identify serum samples containing antibodies to H5 and most importantly, sera containing antibodies to other HA subtypes tested negative for H5N1. Furthermore, similar test results were observed with rH5 HA or whole inactivated H5N1 virions as coating antigens for the EB-ELISA (Fig. 3). Use of recombinant HA antigen provides an attractive safe alternative to H5N1 viral antigen for antibody detection assays.

The relative sensitivity of EB-ELISA and HI assays was further compared by endpoint dilution analysis. Chicken antisera (n = 5) collected 10 days after first immunization with 26 H5N1 Indonesian strains (Table 1) tested positive in EB-ELISA with cut-off value of >30% and achieving a mean titer of 52. Sera collected 10 days after the 2^nd^ immunization showed mean endpoint dilution titer of >334.67 in the EB-ELISA. In contrast, HI assays showed very low levels of antibody (HI titer of <12) on day 10 after the 1^st^ immunization, reaching higher levels on day 10 after 2^nd^ immunization (mean HI titer 85). Sera from chickens immunized with HA subtypes other than H5N1 showed no significant inhibition in EB-ELISA. H3N2 immune chicken sera peaked with a mean HI titer of 6 in HI assay against H5N1 antigen on day 10 after the 2^nd^ immunization (Table 4).

### Performance of EB-ELISA relative to microneutralization assay

**Figure 4:**
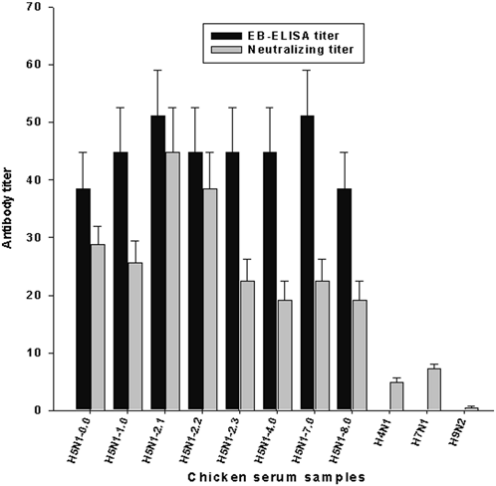
Comparison of EB-ELISA to microneutralization assay. The H5N1 antibody detection sensitivity and specificity of the EB-ELISA and microneutralization assays were compared using immune chicken sera collected 10 days after second immunization (adjusted to HI titer of 16) to determine the endpoint of inhibition. Chicken antisera to clade 0 (A/HK/156/97), 1 (A/Vietnam/1203/04), 2.1 (A/Indonesia/CDC1031/07), 2.2 (A/turkey/Turkey1/05), 2.3 (A/Anhui/1/05), 4 (A/Guiyang/337/06), 7 (A/Shanxi/2/06) and 8 (A/chicken/Henan/12/04) as well as H4N1, H7N1 and H9N2 were used. The EB-ELISA titer was determined as described in the Methods section. The neutralization assays were performed using H5N1 virus A/Indonesia/CDC669/06. The results were expressed as the geometric mean titers (n = 5/group, whiskers above bars represent the standard error of the mean).

The sensitivity of EB-ELISA was also compared to the microneutralization assays which are currently recommended for serological diagnosis of human H5N1 infection. Serum samples from chickens immunized with H5N1 were normalized to HI titer of 16 to the homologous viruses prior to titer determination in the EB-ELISA. H5N1 antisera to viruses from different clades tested positive in EB-ELISA with cut-off value of >30% blocking at a mean end point serum dilution of 44.8 (38.4–51.2). In contrast, serum samples to virus subtypes other than H5N1 tested negative in EB-ELISA (Fig. 4). The clade 2.1 and clade 2.2 H5N1 antisera showed neutralizing antibody titer of 44.8 and 38.4 respectively. H5N1 clade 0, 2.3 and 7 showed neutralizing antibody titers <30 (Fig. 4). Interestingly, serum samples from other clades (clade 1, clade 4 and clade 8) showed neutralizing antibody titers below 20 against H5N1 viruses from clade 2.1 (Fig. 4). In addition, H4N1 and H7N1 antisera yielded negligible neutralizing antibody titers against H5N1 (Fig. 4).

### Human serum samples

To determine whether the 5F8 epitope was consistently recognized by antibodies elicited by natural H5N1 infection in humans, convalescent serum samples from individuals with a laboratory-confirmed H5N1 infection were evaluated in the EB-ELISA. Ten serum samples from patients who had recovered from H5N1 influenza infection tested positive in EB-ELISA performed with mAb 5F8 with mean percent blocking of 73.16 (63.81–83.46%, Table 5). However, these samples showed a low mean HI titer of <16 (8–32 HI titer). Eight of these ten human serum samples showed HI titer of 8–16 and only two samples reached the HI titer of 32 against H5N1 virus from clade 2.1 (Table 5). Sera from patients recovered from seasonal influenza A virus infection yielded negative EB-ELISA results; a maximum blocking of <13% was noted. However, some of the convalescent influenza A human serum samples (4 out of 15 samples) showed HI titer of 8 against H5N1 virus, indicating some degree of cross-reactivity. Normal human sera showed <10% inhibition in the EB-ELISA (Table 5). These data further establish the reliability of EB-ELISA to positively detect H5 antibodies in serum samples from infected hosts without significant false-negative test results due to antibodies elicited by seasonal influenza viruses.

**Table 5:** Analysis of human serum samples in EB-ELISA.

Clinical human serum samples	HI titer against H5N1*	Inhibition in EB-ELISA* (%)
**H5N1 recovered serum**		
Indonesia/16_	16	79.47
Indonesia/292	32	83.46
Indonesia/330	16	76.17
Indonesia/530	16	74.23
Indonesia/554	32	78.37
Indonesia/563	16	78.37
Indonesia/645	16	63.81
Indonesia/650	8	67.14
Indonesia/721	16	65.87
Indonesia/828	16	73.38
**Non-H5 influenza A serum**		
9398	8	12.84
9286	<8	12.56
9216	<8	11.28
9159	<8	6.72
9151	8	11.63
9140	<8	12.83
9115	8	9.48
9129	<8	7.19
9873	<8	8.86
9106	8	9.43
9708	<8	12.16
9824	<8	8.38
9872	8	11.81
9624	<8	12.78
9792	<8	11.29
**Normal human serum**		
n = 20	0.0±0.0 a	9.93%±3.099 a

* Titer expressed relative to neat serum.

a Mean value±S.D of twenty serum samples (*n* = 20).

## Discussion

Active serological surveillance for HPAI H5N1 viruses is crucial for early detection and control of this emerging human pathogen. The conventional subtype-specific methods for serological surveillance are HI, microneutralization assay and ELISA, but these tests have significant limitations. The need for a sensitive and specific serological assay for detection of human antibodies to H5N1 has not been met [26]. Blocking-ELISA offers considerable advantages for detection of viral antibodies. A blocking-ELISA based on a mAb against influenza A nucleoprotein (NP) has been reported as quicker and more sensitive than HI for detection of influenza A virus infection [27]. In addition, EB-ELISA has been used for sensitive and specific detection of antibodies to many other viral pathogens [28]–[32]. In particular, development of a blocking-ELISA for specific detection of antibodies to low pathogenic avian influenza (LPAI) subtype H5N2 viruses was reported recently [33].

The 5F8 mAb selected for this study was found to recognize an epitope including the sequence CNTKCQTP in the HA1 region of the H5 HA. Analysis of this epitope in 4143 influenza A viruses HA sequences from the known subtypes (H1 through H16) revealed that this epitope is highly conserved in H5N1 viruses. The conservation rate of the epitope was absolute (100%) among H5N1 viruses isolated from humans, indicating that this 5F8 mAb-based blocking-ELISA could be used for universal detection of H5N1 antibodies in human sera. The epitope is also highly conserved in H5N1 viruses isolated from avian sources (96.9%). The 5F8 epitope was also found in 54% of low pathogenic avian H5 viruses (H5N2-N9), therefore, the 5F8 mAb-based blocking-ELISA could not distinguish between HPAI H5N1 viruses and low pathogenic H5 subtypes in animals. However, low pathogenic H5 subtype viruses in poultry are also a serious concern due to their propensity to mutate and acquire high virulence. Therefore, detection of H5N1 antibodies using this EB-ELISA can also be especially useful in serological surveillance for H5 virus infection in poultry and wild birds. Of the 2812 non-H5 subtype viruses analyzed, only 14 H1 subtype viruses (2.3%) were found to contain the 5F8 epitope (CNTKCQTP). Interestingly, these H1 subtype viruses were isolated between 1990 and 1999, suggesting that this epitope has become extinct in this subtype. We postulate that the H1N1-elicited 5F8 cross-reactive antibodies that could interfere in the H5 EB-ELISA can be considered relatively low.

The H5 HA genes of HPAI influenza viruses are classified into distinct phylogenic clades and subclades, many of which were targeted for development of H5N1 vaccine candidates [34]. Our data showed that mAb 5F8-based EB-ELISA was able to detect H5N1 antibodies in sera of chickens immunized with H5N1 clades 0, 1, 2.1, 2.2, 2.3, 4, 7 and 8, suggesting that the 5F8 epitope is highly antigenic regardless of the contextual sequence difference between clades. Responses to clade 1, 2.1, 2.2 and 2.3 HA are particularly relevant because these viruses caused the most human infections in Asia, Africa and Europe [25].

Our results suggest that the blocking-ELISA using 5F8 mAb was more effective for detection of H5N1 antibodies than HI and microneutralization assay in terms of sensitivity. Previous studies indicated that the microneutralization assay was the most sensitive method for detection of H5N1 antibodies [26]. However, other reports noted that cross-reacting antibodies from infection with other subtypes limited the specificity of the assay [35]–[37].

Forty-five human serum specimens with known H5N1 exposure history were used to evaluate the performance characteristics of the EB-ELISA. In agreement with chicken antisera results, the 5F8 blocking-ELISA detected H5N1 antibodies in convalescent human sera and yielded no false-positive test with convalescent sera from seasonal influenza virus injections. Further studies are planned to determine whether the EB-ELISA will be useful to measure humoral antibody responses elicited by inactivated H5N1 pre-pandemic vaccines.

In summary, the EB-ELISA developed in this study has significant advantages relative to HI and microneutralization assay for the detection H5N1 antibodies. Consistent blocking of the 5F8 epitope by antibodies sera from chickens immunized with divergent H5 HA clades as well as convalescent human sera highlights the high antigenicity of this epitope. The conservation of this epitope (CNTKCQTP, aa 274–281; equivalent to 277 to 284 in H3 numbering) [38] in influenza H5 HA and its near complete absence in other subtypes indicates that mAb 5F8 meets the critical requirement for diagnosing H5 influenza virus infections using the epitope-blocking assay format. Taken together, these data suggest that this EB-ELISA is an attractive option for detection of antibodies to H5N1 viruses in human and animal sera with potential application for sero-diagnosis and surveillance.

## Supporting Information

Table S1 (0.03 MB DOC)

Table S2 (0.03 MB DOC)

Table S3 (0.03 MB DOC)

Table S4 (0.04 MB DOC)
