# Supplementary material for: Development of Epitope-Blocking ELISA for Universal Detection of Antibodies to Human H5N1 Influenza Viruses
Source: PLoS One. 2009 Feb 24;4(2):e4566. doi: 10.1371/journal.pone.0004566 (PMC2642733; doi:10.1371/journal.pone.0004566)
Supplement: Table S1 — (0.03 MB DOC) [file pone.0004566.s001.doc]

**Table S1:** Primers used for HA gene amplification and cloning

|  | **Sequence** |
| --- | --- |
| Forward primer | 5´-cagagaggatccatggagaaaatag-3´ |
| Reverse primer | 5´-cagagaaagctttcaagggctattt-3´ |
