# Supplementary material for: Development of Epitope-Blocking ELISA for Universal Detection of Antibodies to Human H5N1 Influenza Viruses
Source: PLoS One. 2009 Feb 24;4(2):e4566. doi: 10.1371/journal.pone.0004566 (PMC2642733; doi:10.1371/journal.pone.0004566)
Supplement: Table S2 — (0.03 MB DOC) [file pone.0004566.s002.doc]

**Table S2:** Primers used for step 1 of 5F8 mAb epitope mapping

| **Fragment (amino acid number)** | **Primer pair** |
| --- | --- |
| A (1-59) | Forward primer: 5´-cagagaggatccatggagaaaatag-3´  Reverse primer: 5´-cgatctgtcgactcatccagctacactcacatcatctctca-3´ |
| B (45-119) | Forward primer: 5´-cagagaggatccaatggagtgaagcctctcattttga-3´  Reverse primer: 5´-cgatctgtcgactcatttggggatgatctgaattttctca-3´ |
| C (105-179) | Forward primer: 5´-cagagaggatccttgagcagaaca-3´  Reverse primer: 5´-cgatctgtcgactcagtgaatcccccacagtacta-3´ |
| D (165-239) | Forward primer: 5´-cagagaggatccaataataccaac-3´  Reverse primer: 5´-cgatctgtcgactcagatggcatcattcggctttaaaattgtc-3´ |
| E (225-321) | Forward primer: 5´-cagagaggatccagaatggagttc-3´  Reverse primer: 5´-cgatctgtcgactcaaggggtatttctgagtccagtcgca-3´ |
