# Supplementary material for: Development of Epitope-Blocking ELISA for Universal Detection of Antibodies to Human H5N1 Influenza Viruses
Source: PLoS One. 2009 Feb 24;4(2):e4566. doi: 10.1371/journal.pone.0004566 (PMC2642733; doi:10.1371/journal.pone.0004566)
Supplement: Table S3 — (0.03 MB DOC) [file pone.0004566.s003.doc]

Table S3: Primers used for step 2 of 5F8 mAb epitope mapping

| **Fragment (amino acid number)** | **Primer pairs** |
| --- | --- |
| SF1 (165-254) | Forward primer: 5´-cagagaggatccaataataccaac-3´  Reverse primer: 5´-cagagaaagctttcagtatgcatattctgga-3´ |
| SF2 (165-264) | Forward primer: 5´-cagagaggatccaataataccaac-3´  Reverse primer: 5´-cagagaaagctttcaaattgctgagtcccctt-3´ |
| SF3 (165-274) | Forward primer: 5´-cagagaggatccaataataccaac-3´  Reverse primer: 5´-cagagaaagctttcagcagttaccatattc-3´ |
| SF4 (165-284) | Forward primer: 5´-cagagaggatccaataataccaac-3´  Reverse primer: 5´-cagagaaagctttcacgcccccattggagt-3´ |
| SF5 (165-294) | Forward primer: 5´-cagagaggatccaataataccaac-3´  Reverse primer: 5´-cagagaaagctttcatatgttgtggaatggcat-3´ |
| SF6 (165-304) | Forward primer: 5´-cagagaggatccaataataccaac-3´  Reverse primer: 5´-cagagaaagctttcatttggggcattccccgat-3´ |
| SF7 (165-314) | Forward primer: 5´-cagagaggatccaataataccaac-3´  Reverse primer: 5´-cagagaaagctttcatgcaaggactaatctgt-3´ |
| SF8 (165-321) | Forward primer: 5´-cagagaggatccaataataccaac-3´  Reverse primer: 5´-cagagaaagctttcaagggctattt-3´ |
