# Supplementary material for: Development of Epitope-Blocking ELISA for Universal Detection of Antibodies to Human H5N1 Influenza Viruses
Source: PLoS One. 2009 Feb 24;4(2):e4566. doi: 10.1371/journal.pone.0004566 (PMC2642733; doi:10.1371/journal.pone.0004566)
Supplement: Table S4 — (0.04 MB DOC) [file pone.0004566.s004.doc]

Table S4: Primers used for site-directed mutagenesis in the final step of 5F8 mAb epitope mapping

| **Amino acid substitution** | **Primer pairs** |
| --- | --- |
| G272A | Forward primer: 5´-GAATTGGAATATGCTAACTGCAACACC-3´  Reverse primer: 5´-GGTGTTGCAGTTAGCATATTCCAATTC-3´ |
| N273A | Forward primer: 5´-TTGGAATATGGTGCCTGCAACACCAAG-3´  Reverse primer: 5´-CTTGGTGTTGCAGGCACCATATTCCAA-3´ |
| C274A | Forward primer: 5´-GAATATGGTAACGCCAACACCAAGTGT-3´  Reverse primer: 5´-ACACTTGGTGTTGGCGTTACCATATTC-3´ |
| N275A | Forward primer: 5´-TATGGTAACTGCGCCACCAAGTGTCAA-3´  Reverse primer: 5´-TTGACACTTGGTGGCGCAGTTACCATA-3´ |
| T276A | Forward primer: 5´-GGTAACTGCAACGCCAAGTGTCAAACT-3´  Reverse primer: 5´-AGTTTGACACTTGGCGTTGCAGTTACC-3´ |
| K277A | Forward primer: 5´-AACTGCAACACCGCGTGTCAAACTCCA-3´  Reverse primer: 5´-TGGAGTTTGACACGCGGTGTTGCAGTT-3´ |
| C278A | Forward primer: 5´-TGCAACACCAAGGCTCAAACTCCAATG-3´  Reverse primer: 5´-CATTGGAGTTTGAGCCTTGGTGTTGCA-3´ |
| Q279A | Forward primer: 5´-AACACCAAGTGTGCAACTCCAATGGGG-3´  Reverse primer: 5´-CCCCATTGGAGTTGCACACTTGGTGTT-3´ |
| T280A | Forward primer: 5´-ACCAAGTGTCAAGCTCCAATGGGGGCG-3´  Reverse primer: 5´-CGCCCCCATTGGAGCTTGACACTTGGT-3´ |
| P281A | Forward primer: 5´-AAGTGTCAAACTGCAATGGGGGCGATA-3´  Reverse primer: 5´-TATCGCCCCCATTGCAGTTTGACACTT-3´ |
| M282A | Forward primer: 5´-TGTCAAACTCCAGCGGGGGCGATAAAC-3´  Reverse primer: 5´-GTTTATCGCCCCCGCTGGAGTTTGACA-3´ |
| G283A | Forward primer: 5´-CAAACTCCAATGGCGGCGATAAACTCT-3´  Reverse primer: 5´-AGAGTTTATCGCCGCCATTGGAGTTTG-3´ |
| A284G | Forward primer: 5´-ACTCCAATGGGGGGGATAAACTCTAGT-3´  Reverse primer: 5´-ACTAGAGTTTATCCCCCCCATTGGAGT-3´ |
